# Detecting Neurodevelopmental Toxicity of Domoic Acid and Ochratoxin A Using Rat Fetal Neural Stem Cells

**DOI:** 10.3390/md17100566

**Published:** 2019-10-04

**Authors:** Santokh Gill, V. M. Ruvin Kumara

**Affiliations:** Regulatory Toxicology Research Division, Health Products and Food Branch, Tunney’s Pasture, Health Canada, 251 Sir Frederick Banting Driveway, Ottawa, ON K1A 0K9, Canada; ruvin@vidanamadura.net

**Keywords:** stem cell differentiation, cytotoxicity, neurotoxicity, domoic acid, ochratoxin A, oligodendrocytes, astrocytes, neurons

## Abstract

Currently, animal experiments in rodents are the gold standard for developmental neurotoxicity (DNT) investigations; however, testing guidelines for these experiments are insufficient in terms of animal use, time, and costs. Thus, alternative reliable approaches are needed for predicting DNT. We chose rat neural stem cells (rNSC) as a model system, and used a well-known neurotoxin, domoic acid (DA), as a model test chemical to validate the assay. This assay was used to investigate the potential neurotoxic effects of Ochratoxin A (OTA), of which the main target organ is the kidney. However, limited information is available regarding its neurotoxic effects. The effects of DA and OTA on the cytotoxicity and on the degree of differentiation of rat rNSC into astrocytes, neurons, and oligodendrocytes were monitored using cell-specific immunofluorescence staining for undifferentiated rNSC (nestin), neurospheres (nestin and A2B5), neurons (MAP2 clone M13, MAP2 clone AP18, and Doublecortin), astrocytes (GFAP), and oligodendrocytes (A2B5 and mGalc). In the absence of any chemical exposure, approximately 46% of rNSC differentiated into astrocytes and neurons, while 40% of the rNSC differentiated into oligodendrocytes. Both non-cytotoxic and cytotoxic concentrations of DA and OTA reduced the differentiation of rNSC into astrocytes, neurons, and oligodendrocytes. Furthermore, a non-cytotoxic nanomolar (0.05 µM) concentration of DA and 0.2 µM of OTA reduced the percentage differentiation of rNSC into astrocytes and neurons. Morphometric analysis showed that the highest concentration (10 μM) of DA reduced axonal length. These indicate that low, non-cytotoxic concentrations of DA and OTA can interfere with the differentiation of rNSC.

## 1. Introduction

The central nervous system (CNS) is a complex structure resulting from an intricately orchestrated sequence of events, which includes cell proliferation, differentiation, migration, synaptogenesis, neurogenesis, neurite and network formation, myelination, apoptosis, and gliogenesis. These stages of development occur sequentially according to different temporal profiles within brain regions, resulting in a heterogeneous pattern of synaptic connectivity [1,2,3,4]. Significant connectivity-based changes occur during development, a process that begins during gestation and continues until adolescence. Brain changes that occur during critical periods of development can result in permanent changes in brain structure and function and must be considered in neurotoxicity testing. As a result of these critical development periods, developing brains are more susceptible to chemical exposure than the adult brain [1,2,3,4]. In addition, children have increased susceptibility to toxic chemicals compared to adults because (a) they have higher exposure to chemicals for their body weight; (b) their metabolic pathways are immature, and hence, they are either unable and/or metabolize toxins at a lower rate; (c) the complexity of early childhood development creates “windows of vulnerability”; and (d) children may have more time than most adults to develop chronic, multistage diseases that may have been triggered by exposures early in life. An increased prevalence of neurodevelopmental disorders such as attention deficit, decrease in cognitive functions, or autism in children has been observed over the last four decades. The increase in these disorders is probably a combination of genetic, biological, psychosocial, and environmental risk factors. Developmental exposure to chemicals has been suspected as a potential causal factor [1,2,3,4], and therefore, the early detection of neurotoxicity during development is crucial in preventing long-lasting effects at all stages of life [4,5,6].

Currently, there is limited information on developmental neurotoxicity (DNT), leaving thousands of environmental chemicals with high uncertainty concerning their DNT potential. In vivo methods that are used to evaluate neurotoxicity in developing and/or matured nervous systems are based on neurobehavioral evaluation of cognitive, sensory, and motor functions, accompanied by neuropathological studies. Unfortunately, these methods are too resource intensive concerning time, money, and number of animals. Due to the need to test a large number of chemicals for regulatory requirements, there is a need to develop and validate alternative test strategies which are more rapid, economically feasible, and have an acceptable predictive capacity [7,8]. 

Previously immortalized cell lines, mature neuronal cells, and/or stem cells derived from various cell types have been used for neurotoxicological studies [5,6,9,10,11,12,13]. However, these models do not necessarily reflect physiological differentiation processes involved in neurogenesis, astrocytogenesis, or oligodendrocytogenesis. To overcome this, we chose rat neural stem cells (rNSC) as a model system. Stem cell assays enable the use of dose ranges that are measured in humans in a high-throughput mode in which the effects of multiple chemicals can be tested simultaneously at much lower costs. Furthermore, these types of assays can also permit mode of action-based DNT testing. Domoic acid (DA) was selected as our model test chemical to validate our novel rNSC in vitro assay system for screening chemicals with potential neurological effects. This assay was also used to investigate the potential neurotoxic effects of well-known nephrotoxin, Ochratoxin A (OTA). 

DA is a well-known neurotoxin that is produced by blooms of different species of *Pseudo-nitzschia* and other marine organisms, such as the red alga *Chondria armata* [14,15]. This toxin is transferred through marine food webs and accumulates in seafood products during harmful algal blooms [14,15,16]. DA has been associated with outbreaks of amnesiac shellfish poisoning in humans and with deaths of a variety of sea birds and mammals after algal blooms. The neurotoxic effects of DA are well established in adults and the developing brain. DA exerts its effect through the alteration of neurogenesis, particularly within the hippocampus [17]. OTA is a mycotoxin produced by *Aspergilli* and *Penicillia* molds. As well, it is produced by marine fungus, especially *Aspergillus* species [18,19]. OTA is a potent nephrotoxin in adults and juvenile animals [20] and, to a lesser extent, it is known to be hepatotoxic, embryotoxic, teratogenic, neurotoxic, immunotoxic, genotoxic, and carcinogenic [18,19,20,21]. The information currently available regarding neurotoxic effects exerted by OTA is limited, although some studies have indicated that OTA may affect the developing brain, including altering neurogenesis [9,22]. 

Currently, there are no studies delineating the effects of DA and OTA on the cytotoxicity and differentiation of rNSC into astrocytes, oligodendrocytes, and neurons. Hence, in the present study, we tested the suitability of rNSC neurosphere suspension, attached rNSC neurosphere, and rNSC monolayer systems to determine the appropriate assay model to investigate cytotoxicity [23,24] and degree of differentiation. Furthermore, we conducted a comprehensive morphological analysis on the number of mature differentiated cells with axons, dendrites and branched dendrites, axons length, and Sholl analysis.

## 2. Results

### 2.1. rNSC Proliferation Without Differentiation

**Figure 1:**
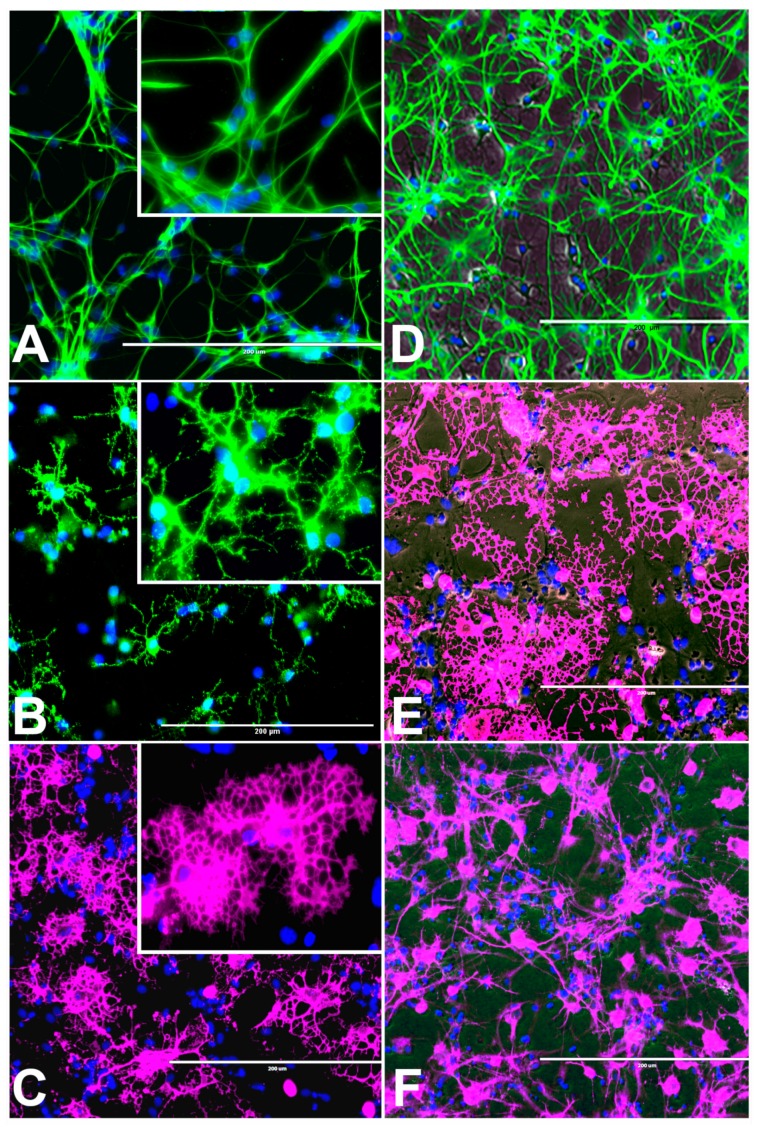
Undifferentiated control rat neural stem cells (rNSC) and rNSC differentiated into oligodendrocytes, astrocytes, and neurons after 7 days in their respective differentiation process. (**A**) Control rNSC cultured in rat neural stem cell culture medium, then immune-stained with neural stem cell-specific marker Nestin (green). (**B**) rNSC cultured in oligodendrocyte differentiation medium, immune-stained with oligodendrocyte-specific markers A2B5 (green). (**C**) Oligodendrocytes immuno-stained with oligodendrocyte-specific marker Galactocerebroside (pink). (**D**) rNSC cultured in astrocyte directed differentiation medium and then immune-stained with astrocyte-specific marker GFAP (green). (**E**) Phase contrast-fluorescent overlapped image of mature oligodendrocytes. (**F**) rNSC cultured in neuron directed differentiation medium, immuno-stained with neuron-specific marker MAP2 clone AP18 (pink). Nucleus marker DAPI (blue). Scale bar indicates 200 μm at 20× magnification.

Characteristic morphology of undifferentiated rNSC is shown after 9 days in complete StemPro NSC SFM medium. This was confirmed by immunofluorescent staining of the rNSC specific marker nestin in the undifferentiated stem cells at day 9 (Figure 1A). Nestin is a well characterized NSC cell marker that is not expressed by mature oligodendrocytes and astrocytes [23].

### 2.2. Neurosphere Assay

#### 2.2.1. Neurosphere Proliferation Without Differentiation

**Figure 2:**
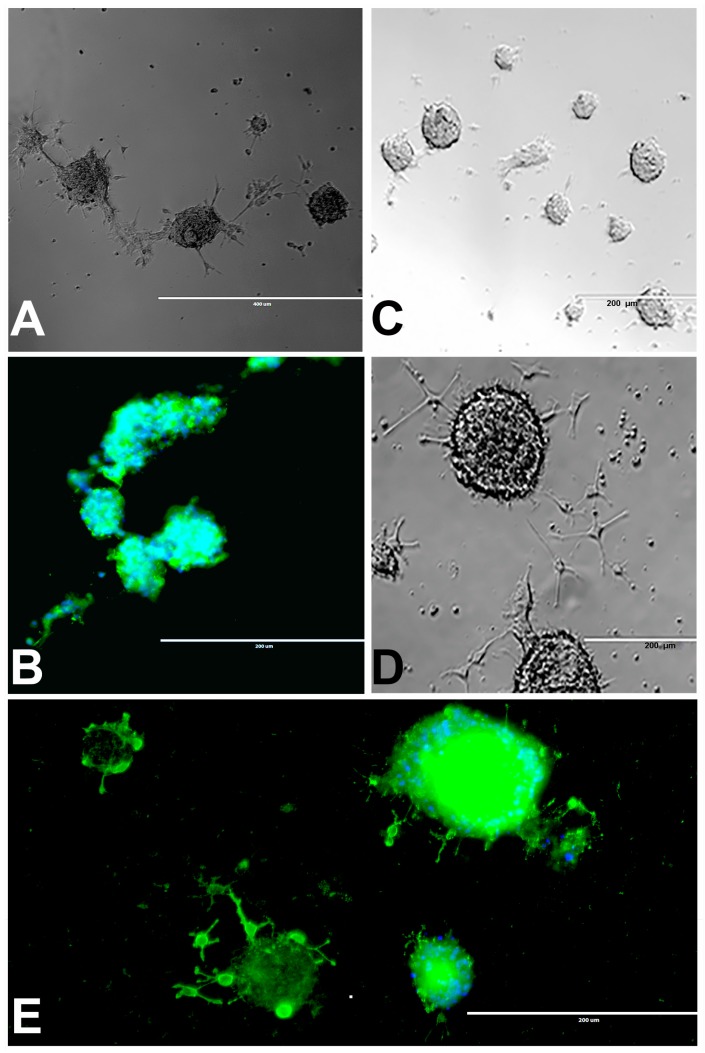
rNSC neurospheres cultured in complete StemPro NSC SFM medium without differentiation factors, or with oligodendrocyte differentiation medium. (**A**) Neurospheres cultured in slide chambers coated with Geltrex showed fusing neurospheres. (**B**) Neurospheres cultured as a suspension in uncoated slide chambers showed fused and fusing neurospheres that were stained with NSC marker nestin. (**C**) Neurospheres cultured in oligodendrocyte directed differentiation medium on day 2, and (**D**) on day 4 of the experiment, showing immature oligodendrocytes coming out from the neurospheres in phase contrast images. (**E**) Day 9 fluorescent image, after staining with oligodendrocyte-specific marker A2B5 (green), showing immature oligodendrocytes coming out from the neurospheres, and nucleus marker DAPI (blue) confirm fully differentiated oligodendrocytes. Scale bar indicates 200 μm or 400 μm at 20× magnification.

We observed the different size of neurospheres ranging from 0.05 to >1 mm in diameter and many of them were fused to form large neurospheres within 2–10 days of culturing in both attached (Figure 2A) and suspension neurospheres (Figure 2B). After 10 days of suspension and immunostaining with rNSC marker, attached neurospheres appeared clearly fused (Figure 2A,B). Our observations are similar to those previously reported [24,25,26]. 

#### 2.2.2. Neurosphere Differentiation into Oligodendrocytes

Most of the rNSC neurospheres attached to Geltrex-coated slide chambers were differentiated into oligodendrocytes (Figure 2C–E). The cell morphology was characterized by bipolar and multipolar morphology (Figure 2D,E) at day 4 and day 9, respectively. After immunostaining with the oligodendrocyte marker, differentiated neurospheres clearly showed fused and fusing morphology (Figure 2B,E).

### 2.3. rNSC Monolayer-Based Models for Three Types of Differentiation Processes

For the monolayer-based system, rNSC were cultured onto chambered slides with cell-specific coating matrix and containing cell-specific directed differentiation media as described in the materials and methods. 

#### 2.3.1. rNSC Differentiation Directed into Oligodendrocytes

The rNSC cultured on poly-l-ornithine and laminin coated chamber slides with oligodendrocyte differentiating medium were differentiated into oligodendrocytes (Figure 1B–E). Of the total number of cells counted, 40% of these cells were oligodendrocytes, as shown by morphology and specific antibody staining. Morphological differences were observed in phase contrast microscope, A2B5 and mGalc immunofluorescent, and DAPI overlapped images (Figure 1B–E). Both A2B5 and mGalc gave comparable results. Differentiation into oligodendrocytes is characterized by bipolar, multipolar, and highly branched neurites in morphology, and networks with neurons and other cells. 

#### 2.3.2. rNSC Differentiation Directed into Astrocytes

**Figure 3:**
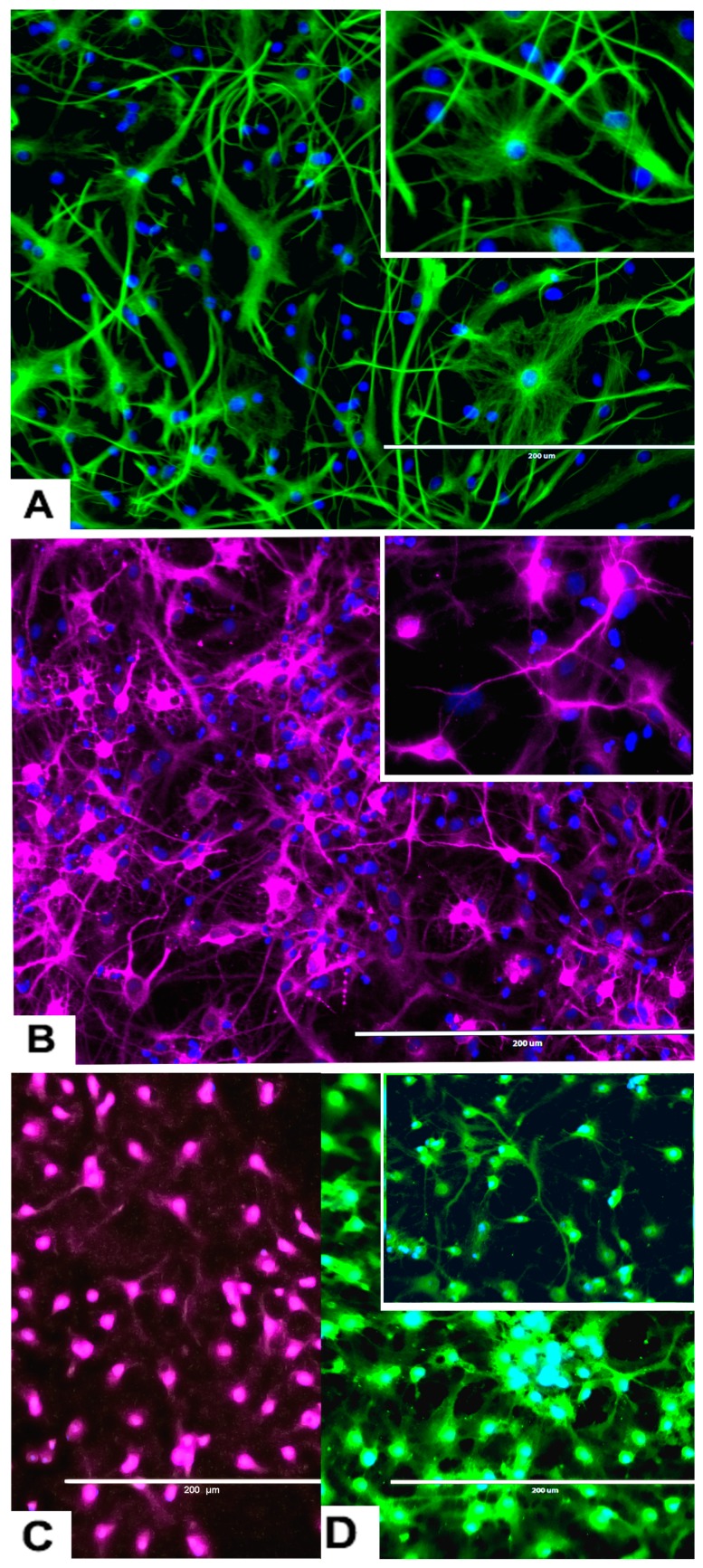
Differentiation of rNSC into astrocytes and neurons after 7 days of the differentiation process. (**A**) rNSC grown in astrocyte differentiation media were differentiated into astrocytes, immuno-stained with astrocyte-specific marker GFAP (green). (**B**) rNSC grown in neuron differentiation media were differentiated into neurons, immuno-stained with neuron-specific marker MAP2 clone AP18 (pink). (**C**) rNSC grown in neuron differentiation media were immuno-stained with neuron-specific marker Doublecortin (purple), and (**D**) MAP2 clone M13 (green). Nucleus marker DAPI (blue). Scale bar indicates 200 μm at 20× magnification.

**Figure 4:**
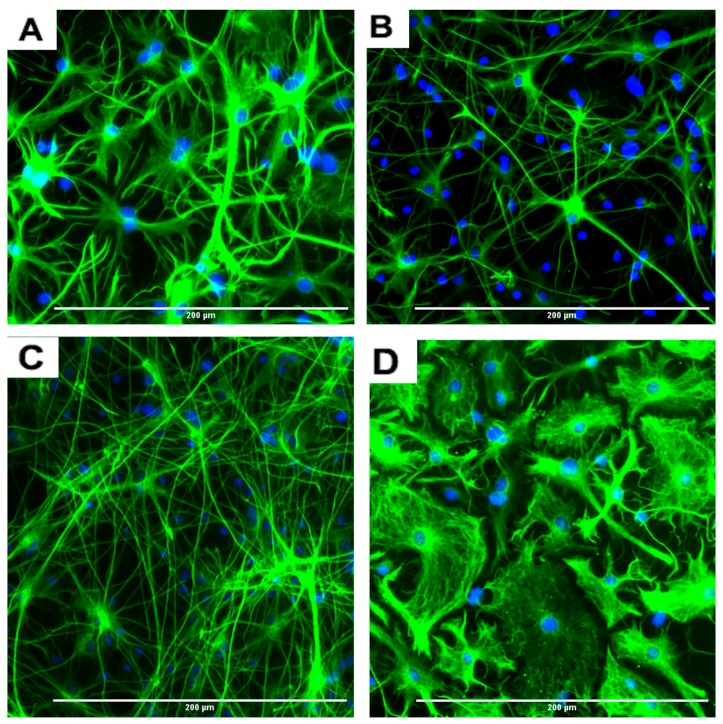
Representative images of four different types of astrocytes observed after the differentiation process for 7 days, in GFAP immunofluorescent and DAPI stained slides. (**A**) Protoplasmic (**B**) fibrous (**C**) varicose projection and (**D**) interlaminar. Scale bar indicates 200 μm at 20× magnification.

The rNSC were cultured in Geltrex-coated chamber slides with astrocyte differentiation media containing the N-2 supplement. Morphological characteristics of astrocytes appeared within 9 days (Figure 1D, Figure 3A, and Figure 4A–D), as observed by the intricate networks associated with astrocytes, and visualized by immunofluorescent staining of astrocyte cell-specific marker GFAP (Figure 1D, Figure 3A, and Figure 4A–D). In the controls, the percentage of differentiation into astrocytes was 46% of the total cells. 

**Figure 5:**
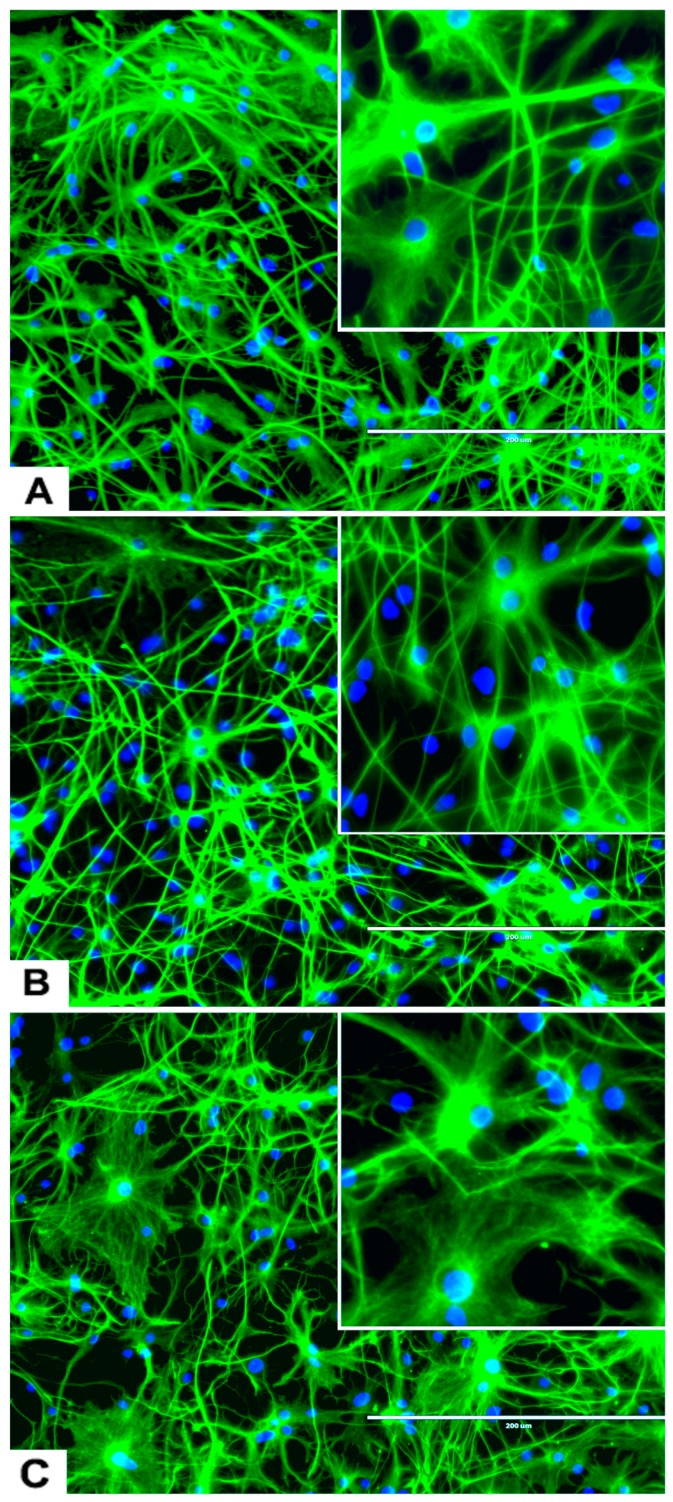
Effects of different concentrations of DA on the differentiation of rNSC into astrocytes. rNSC cultured in astrocyte directed differentiation media with and without DA for 7 days. Mature astrocytes were stained with specific marker GFAP and nucleus marker DAPI. Representative fluorescent images of astrocyte differentiation (**A**) without DA (control), (**B**) with 0.05 μM of DA, and (**C**) with 5 μM of DA, are depicted. Scale bar indicates 200 μm at 20× magnification.

An interesting observation was that four different types of astrocytes were observed in the phase contrast microscope, GFAP immunofluorescent, and DAPI overlapped images. However, only two types, the protoplasmic (Figure 4A) and fibrous (Figure 4B) types have been reported in rodents. The other two types of astrocytes were the varicose projection (Figure 4C) and the interlaminar (Figure 4D) types. These varicose projection-type astrocytes (Figure 4C) and interlaminar-type astrocytes (Figure 4D) have only been reported in higher primates and humans [27,28,29]. The interlaminar astrocytes have spherical cell bodies, whereas the varicose projection astrocytes have long processes. These were observed using immunofluorescence staining with GFAP (Figure 4C,D and seen in Figure 3A and Figure 5A). 

#### 2.3.3. rNSC Differentiation Directed into Neurons

**Figure 6:**
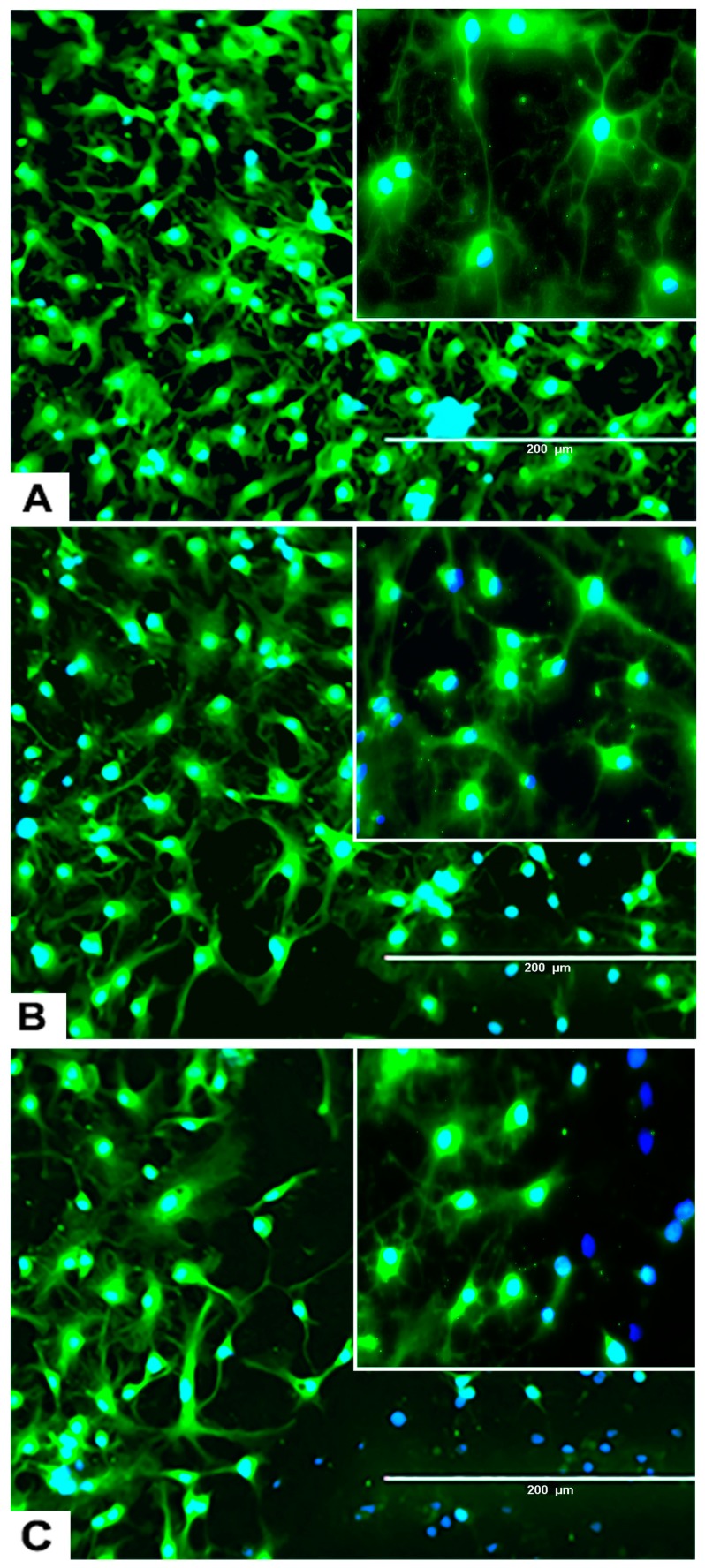
Effects of different concentrations of DA on the differentiation process of rNSC into neurons. rNSC were cultured in neuron differentiation media with and without DA for 7 days. Cells were stained with the neuron-specific marker MAP2 and the nucleus marker DAPI. Representative fluorescent images of neuron differentiation (**A**) without DA (control), (**B**) with 0.05 μM of DA, and (**C**) with 5 μM of DA are depicted. Scale bar indicates 200 μm at 20× magnification.

The rNSC cultured on poly-l-ornithine and laminin coated chamber slides with neuron differentiation medium were differentiated into neurons as manifested by the multipolar morphology and intricate neurite networks (Figure 1F, Figure 3B–D, and Figure 6A). Neurons gradually developed into mature morphology, as observed with a cell body containing a nucleus and extending two types of cytoplasmic dendritic processes. Neurons with several types of axon morphologically including unipolar, bipolar, multipolar, and pyramidal-like neurons were observed in vitro in phase contrast, MAP2 clone AP18 (Figure 1F and Figure 3B), Doublecortin (Figure 3C), and MAP2 clone M13 (Figure 3D) immunofluorescent and DAPI overlapped images. In the controls, the percentage of differentiation into neurons was 46% of the total cells. 

#### 2.3.4. Effects of Domoic Acid on rNSC Differentiation Directed into Astrocytes, Neurons, and Oligodendrocytes 

**Figure 7:**
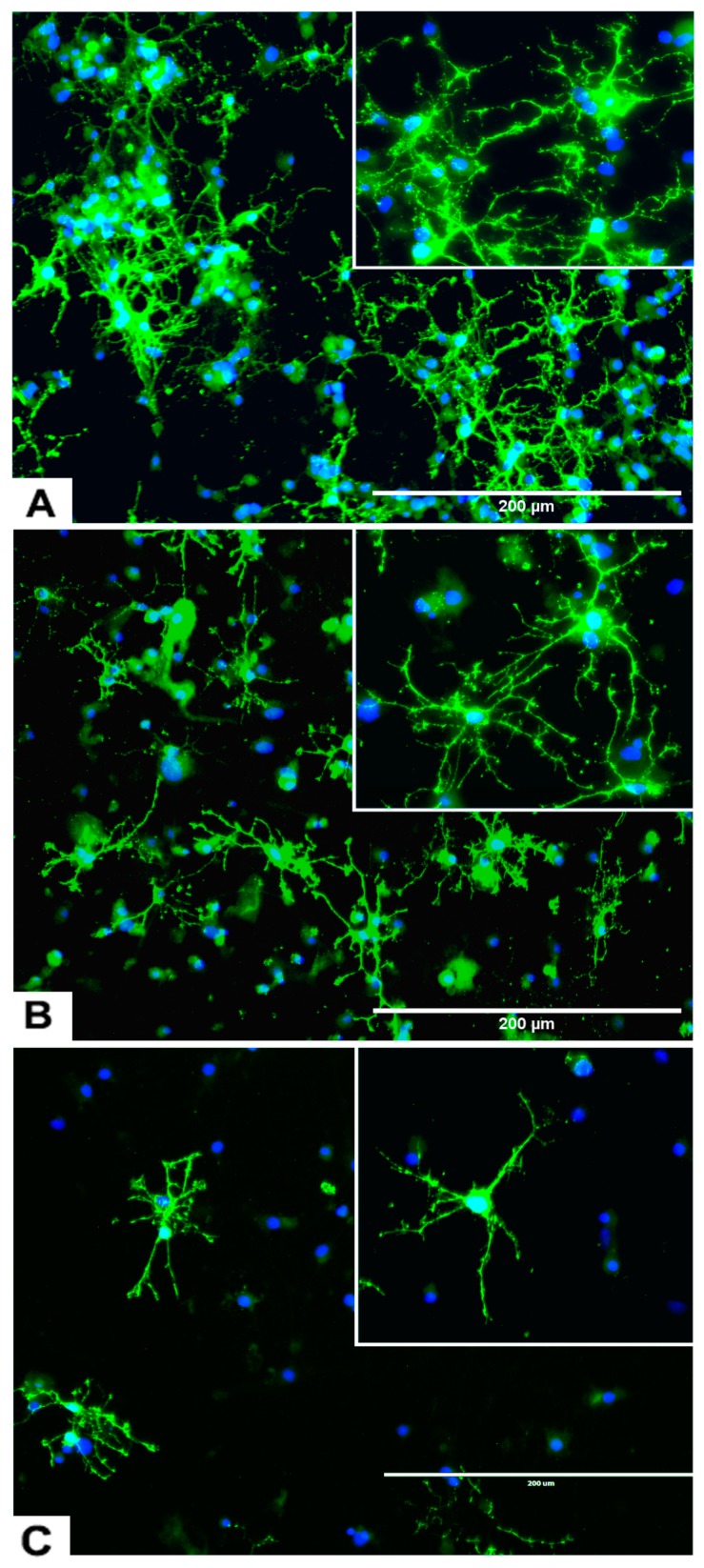
Effects of different concentrations of DA on the differentiation of rNSC into oligodendrocytes. rNSC cultured in oligodendrocyte differentiation media with and without DA for 7 days. Mature oligodendrocytes were stained with specific marker A2B5 and nucleus marker DAPI. Representative fluorescent images of oligodendrocyte differentiation (**A**) without DA (control), (**B**) with 0.05 μM of DA, and (**C**) with 5 μM of DA are depicted. Scale bar indicates 200 μm at 20× magnification.

rNSC were cultured in specific differentiation media and treated as described in the Materials and Methods section. Subsequently, these cells were fixed and immuno-stained with their corresponding cell-specific markers—GFAP for astrocytes, MAP2 and doublecortin for neurons, and A2B5 and mGalc for the oligodendrocytes. Fluorescent images of each differentiated cell type showed a reduction in the number of differentiated cells at day 9 in 0.05 μM of DA (Figure 5B, Figure 6B, and Figure 7B) and 5 μM of DA (Figure 5C, Figure 6C, and Figure 7C), compared to controls in 0 μM of DA (Figure 5A, Figure 6A, and Figure 7A). The reduced number of differentiated cells are shown for astrocytes (Figure 5B,C), neurons (Figure 6B,C), and oligodendrocytes (Figure 7B,C), respectively, compared to their corresponding controls (Figure 5A, Figure 6A, and Figure 7A). 

### 2.4. Effects of DA and OTA on Cytotoxicity and on the Differentiation of rNSC Directed into Astrocytes 

**Figure 8:**
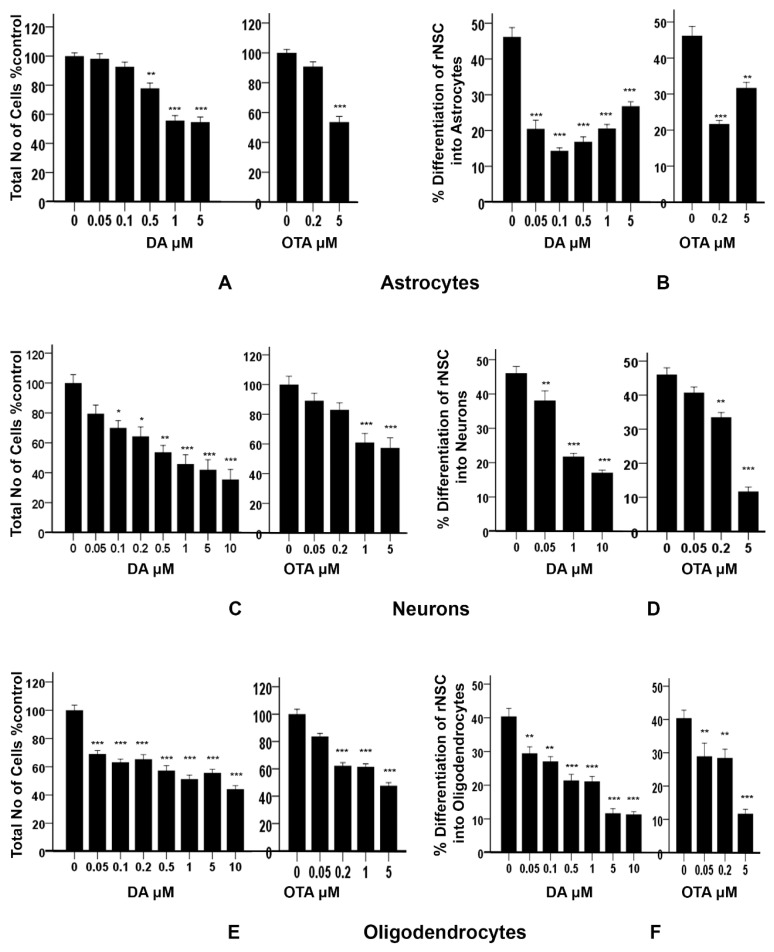
Effects of different concentrations of DA and OTA treated for 7 days on cytotoxicity and percentage differentiation of rNSC in relevant directed differentiation medium. Relative cell count was expressed as “total number of cells % control”, and used as an index of cytotoxicity in (**A**) astrocyte, (**C**) neuron, and (**E**) oligodendrocyte differentiation medium, respectively. Percentage differentiation of rNSC into (**B**) astrocytes, (**D**) neurons, and (**F**) oligodendrocytes after 7 days of differentiation process are shown. Differentiated astrocytes, neurons, and oligodendrocytes stained with specific marker GFAP, MAP2, A2B5 or mGalc, respectively. Morphology with neurites in fluorescent, DAPI, and phase contrast overlapping images were used for the quantification. Percentage differentiation = (No. of nuclei of differentiated cells ÷ number of nuclei of total cells) × 100. Data shown are mean ± SE; * *p* < 0.05, ** *p* < 0.01, *** *p* < 0.001 compared to the control. One-way ANOVA with Tukey’s HSD post-hoc test for cytotoxicity (equal sample size) and Scheffe post-hoc test for percentage differentiation (unequal sample size) were used for multiple comparisons.

The “total number of cells % control” was used as an index of cytotoxicity and the percentage differentiation was used as an index of the degree of differentiation of rNSC into astrocytes, neurons, and oligodendrocytes. There was a statistically significant effect of DA concentration on the percentage of viable cells, F(5,30) = 40.643, *p* < 0.001, as determined by one-way ANOVA. Pairwise comparison of the concentration–response of DA by Tukey’s HSD post-hoc test revealed that all the concentrations of DA except 0.05 μM and 0.1 μM significantly reduced the “total number of cells % control” when compared with the control (Figure 8A). There was no statistically significant difference in the survival rate at the lowest concentrations of DA (0.05 μM and 0.1 μM) compared to controls. Concentration–response graph of DA on “total number of cells % control” clearly showed that the concentration of DA is directly proportional to the cytotoxicity. Similarly, OTA showed a significant reduction in the “total number of cells % control” F(2,15) = 62.845, *p* < 0.001. Tukey’s HSD post-hoc test revealed that 5 μM of OTA significantly reduced the “total number of cells % control” (*p* < 0.001), while 0.2 μM of OTA did not reduce it when compared with the control (Figure 8A). The data suggest that DA concentrations of 0.5 μM or greater and 5 μM of OTA are cytotoxic.

In addition, these data showed a statistically significant effect of DA concentration on the percentage differentiation of rNSC into astrocytes F(5,53) = 43.696, *p* < 0.001. Scheffe post-hoc tests revealed that all DA concentrations significantly reduced the percentage differentiation of rNSC into astrocytes in a concentration-dependent manner (Figure 8B). Even DA concentrations that were shown to be non-cytotoxic (0.05 μM and 0.1 μM) significantly reduced (*p* < 0.001) the differentiation of rNSC into astrocytes compared to control, 25.73 ± 2.59% and 31.86 ± 2.68%, respectively (Figure 5A,B and Figure 8B). 

Similarly, OTA-treated cells showed a significant effect on the percentage differentiation of rNSC into astrocytes F(2,32) = 44.198, *p* < 0.001. Only 0.2 μM OTA and greater treated cells showed a significant reduction in percentage differentiation of rNSC into astrocytes (*p* < 0.001), while 0.2 μM OTA-treated rNSC did not show significant reduction in “total number of cells % control” (Figure 8A,B). 

### 2.5. Effects of DA and OTA on Cytotoxicity and on the Differentiation of rNSC Directed into Neurons 

There was a statistically significant effect of different concentrations of DA on the “total number of cells % control” F(7,40) = 13.335, *p* < 0.001, as tested by one-way ANOVA. Tukey’s HSD post-hoc test revealed that all concentrations except 0.05 μM of DA significantly reduced the “total number of cells % control” when compared with the control (Figure 8C). The concentration–response graph of DA on “total number of cells % control” clearly showed that DA increased the cytotoxicity in a dose-dependent manner. 

Similarly, different concentrations of OTA-treated cells showed statistically significant effects on the “total number of cells % control” F(4,25) = 10.423, *p* < 0.001. Tukey’s HSD post-hoc test showed that only 1 μM and 5 μM of OTA significantly reduced “total number of cells % control” and hence increased cytotoxicity compared to the control. However, 0.05 μM and 0.2 μM of OTA-treated cells did not show statistically significant differences compared with the control. DA- and OTA-treated cells showed a significant increase in cytotoxicity in the concentration–response graph. It showed that the number of cells were significantly reduced at 0.1 μM and greater concentrations of DA, and 1 μM and greater concentrations of OTA compared to controls (Figure 8C). The concentration of 0.05 μM of DA, and 0.05 μM and 0.2 μM of OTA, were non-cytotoxic. 

Different concentrations of DA showed statistically significant effects on the percentage differentiation of rNSC into neurons F(3,66) = 65.560, *p* < 0.001. Scheffe post-hoc test showed that all DA concentrations significantly reduced the percentage differentiation of rNSC into neurons (Figure 8D). 

Similarly, different concentrations of OTA showed statistically significant effects on the percentage differentiation of rNSC into neurons F(3,52) = 65.305, *p* < 0.001. Scheffe post-hoc test showed that only 0.2 μM and 5 μM of OTA significantly reduced the percentage differentiation of rNSC into neurons compared to the control. Whereas, 0.05 μM of OTA did not significantly reduce the percentage differentiation of rNSC into neurons (Figure 8D). Although there was no effect on the survival of total cells starting at 0.05 μM of DA and 0.2 μM of OTA (Figure 8C), there was a statistically significant reduction on the differentiation of neurons (Figure 8D, also seen in Figure 6A,B) compared to controls.

The degree of percentage differentiation of rNSC into neurons was decreased with increasing concentrations of DA (Figure 6A–C and Figure 8D) or OTA (Figure 8D) when compared with controls. 

### 2.6. Effects of DA and OTA on Cytotoxicity and on the Differentiation of rNSC Directed into Oligodendrocytes

Cytotoxicity studies showed statistically significant effects of different concentrations of DA on the “total number of cells % control” F(7,42) = 33.822, *p* < 0.001, as determined by one-way ANOVA. Pairwise comparison of the DA-treated groups by Tukey’s HSD post-hoc test revealed that all the used concentrations of DA significantly reduced the “total number of cells % control” (*p* < 0.001) when compared with the control (Figure 8E). The concentration–response graph of DA revealed that all groups of DA-treated cells showed cytotoxicity. 

Similarly, OTA-treated groups showed statistically significant effects on the “total number of cells % control” F(4,25) = 68.304, *p* < 0.001. Pairwise comparison of OTA-treated groups by Tukey’s HSD post-hoc test revealed that all the concentration of OTA-treated groups except 0.05 μM of OTA significantly reduced the “total number of cells % control” (*p* < 0.001). 

It showed that all concentrations of DA and OTA tested significantly reduced the “total number of cells % control” except 0.05 μM of OTA (Figure 8E). This indicates that all concentrations of DA and OTA tested are cytotoxic except 0.05 μM of OTA, suggesting that oligodendrocyte differentiation may not be susceptible to low concentrations of OTA. 

The data also showed a statistically significant effect of different concentrations of DA on the percentage differentiation of rNSC into oligodendrocytes F(6,84) = 35.848, *p* < 0.001. Pairwise comparison of all DA-treated groups by Scheffe post-hoc tests showed a significant reduction in percentage differentiation of rNSC into oligodendrocytes in DA-treated groups compared to the control (Figure 8F). All cytotoxic concentrations of DA (Figure 8E) significantly reduced the degree of differentiation of rNSC into oligodendrocytes compared to the control (Figure 7A–C and Figure 8F). Immunostaining for oligodendrocytes showed that exposure to DA also reduced the degree of differentiation of rNSC into oligodendrocytes with increasing concentration of DA (Figure 7A–C).

Similarly, there was a statistically significant effect of different concentration of OTA on the percentage differentiation of rNSC into oligodendrocytes, F(3,44) = 20.499, *p* < 0.001. Scheffe post-hoc test showed a significant reduction in percentage differentiation of rNSC into oligodendrocytes in OTA-treated groups compared to the control (Figure 8F).

A dose–response graph showed that all concentrations of both DA and OTA significantly reduced the percentage differentiation of rNSC into oligodendrocytes relative to controls (Figure 8F). However, the non-cytotoxic concentration of 0.5 μM OTA also significantly reduced the percentage differentiation of rNSC into oligodendrocytes. These data suggest that both non-cytotoxic and cytotoxic concentrations have an effect on the differentiation of rNSC into oligodendrocytes.

### 2.7. Effects of DA and OTA on the Axonal Length of the Mature Neurons

**Figure 9:**
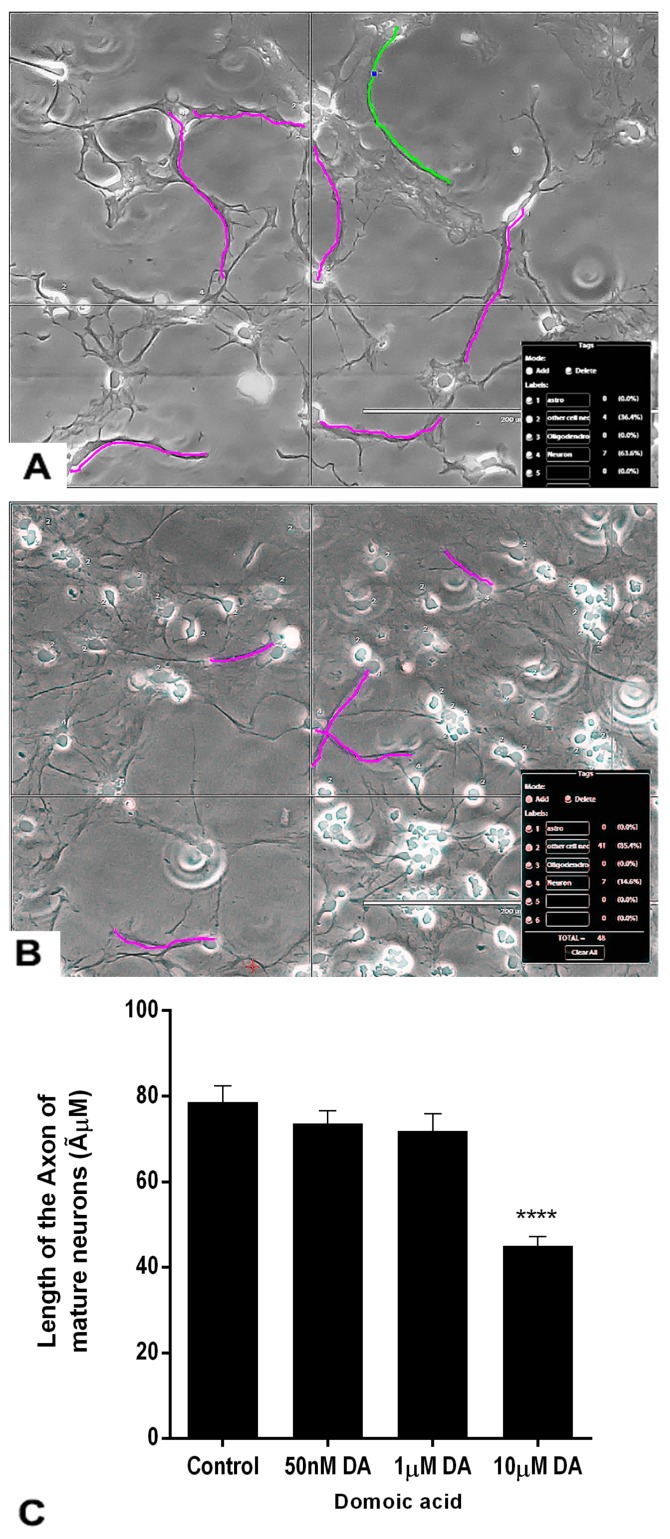
Effects of different concentrations of DA after 7 days of treatment on the length of the axons of neurons. The neurons were differentiated from rNSC in slide chambers with neuron differentiation medium with and without DA. (**A**) Representative image for tracing the axon of neurons to measure the length of control neurons. (**B**) 10 μM DA treated neurons. (**C**) Traced length of the axon of neurons treated with 0 μM (control, *n* = 65), 0.05 μM (*n* = 75), 1 μM (*n* = 71), and 10 μM (*n* = 61) of DA. Data shown are mean ± SE, **** *p* < 0.001 compared to the control, one-way ANOVA with the Kruskal–Wallis H test.

The axon length of the differentiated neurons was measured at the end of the treatment (Figure 9C). The axons were traced in magenta color in luminous images of controls and DA-treated neurons (Figure 9A,B). Non-parametric analysis done by the Kruskal–Wallis H test revealed that there was a statistically significant difference in axonal length of mature neurons between DA-treated groups and the control group, χ^2^ (3) = 58.387, *p* < 0.001. The mean rank axonal length of neurons was 166.95 for control (*n* = 65), 155.74 for 0.05 μM DA (*n* = 74), 141.14 for 1 μM DA (*n* = 71), and 69.78 for 10 μM DA (*n* = 60) treated groups. Only the highest concentration of DA (10 μM) significantly reduced the axonal length of developing mature neurons compared with the controls (Figure 9B,C). Lower concentrations of DA, 0.05 μM and 1 μM, did not significantly change the axonal length of the mature neurons compared with controls. 

Similarly, the above experiment was repeated with OTA to observe any effects on the axonal length of the mature neurons. Visual observations revealed no differences in axonal length of the neurons differentiated at 0.05, 1, or 10 μM of OTA (data not shown). 

## 3. Discussion

CNS neural stem cells present in developing and adult brains are multipotent, and thus able to differentiate into neurons, astrocytes, and oligodendrocytes. Throughout life, neural stem cells are present in the subventricular zone of the lateral ventricles and the subgranular zone of the dentate gyrus of the hippocampus. Hence, it is important to study the effects of toxins on these cells and how they could contribute to neurotoxicity and neurobehavioral outcomes. In this study, we differentiated rNSC into astrocytes, neurons, and oligodendrocytes in the presence and absence of DA or OTA. We tested the suitability of the rNSC suspension neurosphere assay, rNSC attached neurosphere assay, and rNSC monolayer system to investigate cytotoxicity and degree of differentiation. The results of the rNSC suspension neurosphere and attached neurosphere assays were unreliable and unreproducible as fusion of neurospheres were observed. Their quantification did not correlate with the rNSC numbers. These data are in agreement with the published data [24,25,30,31]. These authors have also highlighted the limitations and accuracy of the neurosphere assay for measuring NSC frequency in relation to NSC regulation. Based on these limitations, we abandoned the use of suspension neurosphere and attached neurosphere models. Hence, we used the rNSC monolayer-based system to investigate the effects in the presence and absence of DA and OTA on the cytotoxicity and degree of differentiation of rNSC into oligodendrocytes, astrocytes, and neurons. For the astrocytes and neurons, the differentiation media has been described by others; for differentiation into oligodendrocytes, differentiation medium was supplemented with neurogenic 2% B27 (components are listed in the Materials and Methods section), which is known to enhance maximum in vitro survival, improved maturation, and functionality of pluripotent stem cell (PSC)-derived neurons [32,33,34].

In the absence of any chemical exposure, the percentage of differentiation of rNSC into astrocytes and neurons was approximately 46.0%, and 40.0% for the oligodendrocytes. Non-cytotoxic and cytotoxic doses of DA and OTA resulted in the reduction of differentiation of rNSC into all three cell types, albeit in different ratios. For DA, 0.05 μM was non-cytotoxic for astrocytes and neurons but reduced the degree of differentiation of astrocytes (25.73 ± 2.59%) and neurons (7.95 ± 2.48%) compared with the relevant control. The non-cytotoxic dose for OTA for astrocytes and neurons was 0.2 μM, and much lower for the oligodendrocytes (0.05 μM). However, these doses reduced the degree of differentiation of astrocytes (24.5 ± 2.62%), neurons (12.53 ± 2.62%), and oligodendrocytes (11.47 ± 3.66%), compared with the relevant control. Based on the reduction of the degree of differentiation, DA was more potent than OTA. Furthermore, DA led to a reduction in the axon length at 10 μM (45.00 ± 2.22 ÃμM) compared with the control (78.67 ± 3.72 ÃμM). 

Another noteworthy observation was that the differentiation of rNSC into astrocytes resulted in four types of morphologically distinct astrocytes, namely the protoplasmic, fibrous, varicose projection, and interlaminar types. Only the protoplasmic and fibrous types have been reported in rodents. The varicose projection and interlaminar types have only been reported in higher primates and humans [18,19,23]. This differentiation of rNSC into these two additional types of astrocytes could be attributed to the use of some components in the differentiation media of human origin (human transferrin and recombinant human insulin). We did not quantify or characterize which types of astrocytes was affected by these toxins, as there are a limited number of markers to identify and distinguish potentially heterogeneous astrocyte subtypes in animal models and in the human brain [31]. However, we counted the total number of astrocytes based on GFAP immunostaining.

The results show that both OTA and DA reduced degrees of differentiation of the main cell types of the CNS. This reduction in the mature cell types and changes in the axonal length may lead to changes in the cytoarchitectural or neural network changes in the brain, which may lead to functional/behavioral changes [35]. Alteration of the oligodendrocyte to neuron ratio may affect cognition due to the role of oligodendrocytes in synapse formation [35]. The astrocytes are the most abundant cells, of which the main task is to maintain the physiological homeostasis of neurons by providing antioxidant protection, substrates for neuronal metabolism, and glutamate clearance [36]. In addition, astrocytes are also involved in regulating synaptic activity and neuronal circuitry. Astrocytes are also known to modulate the blood–brain barrier (BBB) development during late embryogenesis and after birth [37]. Given the close coupling between astrocytes and neurons to form the neurovascular unit in adulthood [37], it is likely that its development must also be tightly regulated to coordinate developing cerebral vascular supply to neuronal demand [31]. Therefore, it could be hypothesized that both DA and OTA could interfere with the development of the BBB in the fetus and of neurovascular units in adulthood, since there was a decrease in the degree of differentiation of astrocytes and neurons upon exposure to these toxins. The BBB is formed by cerebral blood vessel endothelial cells in concert with astrocyte endfeet and creates a barrier between blood and the brain parenchyma. In humans, protoplasmic astrocytes wrap around blood vessels much more completely than they do in mice and rats, hence playing a much more important role in keeping agents in the blood from entering the brain and in regulating blood flow [38]. 

Oligodendrocytes are the most vulnerable cells in the brain [39]. This is supported by our findings as well, because the degree of differentiation into oligodendrocytes was the most affected by DA and OTA. Oligodendrocytogenesis during brain development is necessary for proper brain function, as oligodendrocytes form and maintain myelin sheaths around axons in the CNS. Disturbances of oligodendrocyte development may result in demyelination diseases that would severely affect neuronal functioning. These degenerative events could lead to changes in conduction velocity, the formation of redundant myelin, and enhanced thickness of myelin sheaths. Studies have shown that adult neural stem cells differentiated into oligodendrocytes are capable of re-myelinating axons following injury and myelin damage [40,41]. 

DA led to a reduction in the axonal length of neurons. Case reports of DA-poisoned patients show that they suffered from diffuse axonopathy [15], diffuse axonal sensorimotor neuropathy, and diffuse axonal damage [42]. DA poisoning in laboratory animals also showed degenerated axons [43,44]. In addition, DA exerts its neurodevelopmental effects through the alteration of neurogenesis and morphology, particularly within the hippocampus, showing EEG irregularities and decreased threshold to DA-induced seizures [15]. According to previously published data, DA-epileptic disease is characterized by spontaneous recurrent seizures after weeks to months of DA poisoning. Furthermore, atypical behaviors in animals and the latent period of silent toxicity characterizes the transition between DA poisoning and epileptic disease [14,45,46]. 

The data from this study show the reduction in the degree of differentiation of rNSC into all three type of cells and the reduction in axonal length of neurons in the presence of low non-cytotoxic concentration of DA (0.05 μM). Data from rodents and non-human primates have shown that non-cytotoxic low dose exposure to DA during early life was associated with behavioral, memory deficits, and/or structural changes in the brain [47,48,49,50,51,52]. This memory loss may be linked to a reduction and/or the abnormal differentiation of neural stem cells. Several studies have shown that transplanted mouse NSCs differentiate into mature cell types within the brain and improve learning and memory in mouse models of Alzheimer’s disease [53,54,55]. 

The non-cytotoxic concentrations of OTA that reduced the degree of differentiation is consistent with the previous data published by Sava et al [9]. They showed that low concentrations of OTA (0.01–100 µg/ml) caused a dose-dependent decrease in viability of both proliferating and differentiating NSC. However, these authors did not quantify rNSC differentiation into astrocytes, neurons, and oligodendrocytes. Further, non-cytotoxic concentrations of OTA (<10 nM) perturbed the homeostasis of highly differentiated neural stem cell cultures [11]. 

The mechanism by which DA and OTA reduce the degree of differentiation of rNSC into the different cell types is not fully known at present. The cytotoxic effect of these chemicals could be due to the activation of intracellular pathways that lead to apoptosis, even at exposure levels that do not cause the death of other neural cell types. DA is known to activate α-amino-3-hydroxy -5-methyl-4-isoxazolepropionic acid and kainate receptors which, in turn, causes glutamate release that subsequently activates the N-methyl-d-aspartic acid receptor, causing apoptotic and necrotic neuronal cell death [56,57]. The effects of OTA on the developing brain have not been fully characterized [58]. To fully elucidate the mechanisms of action of DA and OTA on neurogenesis, molecular pathways related to the developmental processes need to be deciphered. 

## 4. Materials and Methods 

### 4.1. Reagents

All reagent preparation, use, and disposal of toxic and bio-hazardous materials were performed according to the laboratory safety regulations of Health Canada. Both DA and OTA were purchased from Sigma-Aldrich (St. Louis, MO, USA). These were dissolved in ethanol, and sterile water was added to make a 1 mM stock solution (final concentration of 0.1% ethanol), which was stored at −20 °C until use. Different working dilutions were made in the relevant differentiation medium. T3 (Thyroxin hormone; Sigma-Aldrich, St. Louis, MO, USA) was dissolved in 1 N NaOH and sterile culture medium to make 20 µg/mL stock solution, stored at −20 °C, and used according to the manufacturer’s instruction. Fetal rNSC (Gibco, Life Technologies Corporation, Frederick, MD, USA) and Lab-Tek chamber slide systems (Thermo Fisher Scientific, Waltham, MA, USA) were used for cell culturing and immunofluorescence staining throughout all experiments. 

The primary antibodies, Anti-Nestin antibody (Abcam, Cambridge, MA, USA), Anti-Glial Fibrillary Acidic Protein (GFAP) antibody (Abcam, Cambridge, MA, USA), Anti-A2B5 antibody (Abcam, Cambridge, MA, USA), anti-Galactocerebroside clone mGalc (Galc) antibody (Millipore, MilliporeSigma, Burlington, MA, USA), Microtubule Associated Protein2 (MAP2) clone M13 monoclonal antibody (Invitrogen, Life Technologies Corporation, Carlsbad, CA, USA), MAP2 clone AP18 monoclonal antibody (Invitrogen, Life Technologies Corporation, Carlsbad, CA, USA), and Doublecortin monoclonal Antibody (Invitrogen, Life Technologies Corporation, Carlsbad, CA, USA), were used as markers for neural stem cells, astrocytes, oligodendrocytes, and neurons in immunofluorescence studies. Secondary antibody, Goat Anti-Mouse IgG H&L Alexa Fluor® 488 (Abcam, Cambridge, MA, USA) was used to detect the Anti-Nestin, Anti-A2B5, and MAP2 clone M13 antibodies, whereas Goat anti-Mouse IgG (H + L) secondary antibody Alexa Fluor Plus 647 (Invitrogen, Life Technologies Corporation, Carlsbad, CA, USA) was used to detect Doublecortin monoclonal antibody. Goat Anti-Rabbit IgG H&L Alexa Fluor® 488 (Abcam, Cambridge, MA, USA) was used to detect Anti-GFAP antibody. Mouse IgG (H + L) cross-Adsorbed Alexa Fluor 594 secondary antibody (Invitrogen, Life Technologies Corporation, Carlsbad, CA, USA) was used to detect anti-Galactocerebroside clone mGalc antibody, and MAP2 clone AP18 monoclonal antibody. All the primary and secondary antibodies were diluted in 5% Goat serum. 

### 4.2. Cell Culture

#### 4.2.1. Propagation of rNSC

Rat neural stem cell culture medium (complete StemPro NSC SFM) was composed of 20 ng/mL of recombinant human fibroblast growth factor basic (FGFb, Gibco, Life Technologies Corporation, Carlsbad, CA, USA), 20 ng/mL of recombinant human epidermal growth factor (EGF, Gibco, Life Technologies Corporation, Carlsbad, CA, USA), and 2 mM Glutamax 100×, 2% StemPro Neural Supplement in 1× KnockOut™ D-MEM/F-12 medium (Gibco, Life Technologies Corporation, Carlsbad, CA, USA). The surface of the T75 culture flask was coated with 15 mL of Poly-L-ornithine (20 μg/mL, Sigma, Burlington, MA, USA) working solution and incubated overnight at room temperature (RT), rinsed twice with D-PBS without Ca^2+^ and Mg^2+^, and used for the rNSC culture. Commercially available multipotent fetal rNSC extracted from *Sprague-Dawley* embryonic day 14 rats (Gibco) were differentiated into astrocytes, neurons, and oligodendrocytes [59,60]. rNSC were seeded (2 × 10^6^ cells) in complete StemPro NSC SFM medium and incubated at 37 °C, 5% CO_2_, and 90% humidity. On the following day, the medium was replaced with an equal volume of fresh, pre-warmed complete StemPro NSC SFM. The medium was replaced every 2–3 days. The cells were passaged when culture was at 75–90% confluent; and P3 cells were subsequently used for the differentiation experiments.

#### 4.2.2. rNSC Neurosphere-Based Model for Differentiation

Attached neurospheres and neurosphere suspensions were used for this experiment. For attached neurospheres, each chamber was coated with 400 µL of 1× Geltrex (Gibco, Life Technologies Corporation, Carlsbad, CA, USA) for 4 h at RT in laminar flow and the excess Geltrex solution was removed immediately before use. For neurosphere suspension, uncoated slide chambers were used. rNSC were seeded at 5.6 × 10^4^ cells/chamber in complete StemPro NSC SFM medium and incubated at 37 °C, 5% CO_2_, and 90% humidity. After 2 days, the Geltrex-coated slide chambers showed attached neurospheres, while uncoated slide chambers showed suspension neurosphere culture. Medium in the coated slide chambers was replaced by the oligodendrocyte differentiation medium, whereas medium of the control chambers was changed with same complete StemPro NSC SFM medium. All chamber slides were incubated at 37 °C, 5% CO_2_, and 90% humidity, and relevant media were changed every 2 days and continued culturing for 7 days for the differentiation process. However, when attempts were made to change the media for suspension neurosphere cultures, most of the smaller suspended neurospheres were lost during aspiration. 

#### 4.2.3. rNSC Monolayer-Based Model for Differentiation

##### A. Coating Chamber Slides for Differentiation into Various Cell Types

For monolayer-based assays and for differentiation into neurons and oligodendrocytes, the chamber slides were double coated. First, each chamber surface was covered with 500 µL of Poly-L-ornithine (20 μg/mL) and incubated overnight at RT in laminar flow, and subsequently washed twice with sterile water and air dried. This was followed by coating each chamber with 500 µL of laminin (10 μg/mL, Gibco, Life Technologies Corporation, Frederick, MD, USA) and incubated for 2 h at 37 °C and rinsed with D-PBS without Ca^2+^ and Mg^2+^. 

For astrocytes, each chamber was covered with 100 µL of Geltrex (1×, Gibco) working solution and incubated for 1 h at 37 °C until dry. 

##### B. Differentiating Media

Astrocyte differentiation medium contained 1% N-2 supplement (1 mM of human transferrin, 2 μM of progesterone, 10 mM of putrescine, 86.1 μM of recombinant human insulin, 3 μM of selenite, 100×, Gibco, Life Technologies Corporation, Frederick, MD, USA), 1% FBS (Gibco, Life Technologies Corporation, Frederick, MD, USA), 2 mM Glutamax (100×) in 1× DMEM medium (Gibco, Life Technologies Corporation, Frederick, MD, USA). Neuron differentiation medium contained 2% B27 (50×, Gibco, Life Technologies Corporation, Frederick, MD, USA), 2mM Glutamax (100×) in 1× Neurobasal medium (Gibco, Life Technologies Corporation, Frederick, MD, USA). Oligodendrocyte modified differentiation medium contained 1× DMEM/F12 (Gibco, Life Technologies Corporation, Frederick, MD, USA) supplemented with 1% N-2 supplement (100×, Gibco, Life Technologies Corporation, Frederick, MD, USA), 2% B27 (50×, Gibco), 49 ng/mL of T3 (Sigma), and 1% BSA (Thermo Fisher Scientific, Waltham, MA, USA). The addition of neurogenic 2% B27 is known to enhance the maximum growth in vitro survival, improved maturation, and functionality of PSC-derived neurons (Thermo Fischer Scientific, Waltham, MA USA). Components of B27 include anti-oxidants (catalase, reduced glutathione, superoxide dismutase, dl-alpha-tocopherol, dl-alpha-tocopherol-acetate), hormones (human Insulin, T3, corticosterone, progesterone), human Holo-transferrin, retinol acetate, putrescine, biotin, l-carnitine, ethanolamine, d(+)-galactose, sodium selenite, linoleic acid, linolenic acid, oleic acid, and pipecolic acid [61].

##### C. rNSC Culturing for Differentiation

rNSC (P3) were seeded at 5.6 × 10^4^ cells per chamber in complete StemPro NSC SFM medium in all chambers and incubated at 37 °C, 5% CO_2_, and 90% humidity. The cells were allowed to attach for 2 days, the, medium in the control chambers coated with poly-l-ornithine was replaced by the complete StemPro NSC SFM medium for culturing rNSC without differentiation. For the differentiation of rNSC to astrocytes, medium in the slide chambers coated with Geltrex was replaced with astrocyte differentiation medium. For the differentiation of rNSC into oligodendrocytes, medium in the slide chambers coated with poly-l-ornithine and laminin was replaced with oligodendrocyte differentiation medium. For the differentiation of rNSC to neurons, medium in the slide chambers coated with poly-l-ornithine and laminin was replaced with neuron differentiation medium. All chamber slides were incubated at 37 °C, 5% CO_2_, and 90% humidity and relevant media were changed every 2 days and continued culturing for 7 days for the differentiation process. After 7 days (DIV 9–10) of differentiation, process medium in each chamber was aspirated and cells were washed once with PBS and aspirated. To fix the cells, 0.5 mL of 4% PFA was added and incubated at RT for 15 min, and washed three times with PBS with Ca^2+^ and Mg^2+^. Then, 0.5 mL of PBS with Ca^2+^ and Mg^2+^ was added to each chamber, wrapped with parafilm, and stored at 4 °C for immunostaining.

### 4.3. Cytotoxicity Assays (Relative Cell Count)

The relative cell count (RCC) was expressed as a “total number of cells % control” and was used as an index of cytotoxicity, which has been used in previous publications [62,63].

### 4.4. Effects of DA and OTA on Directed Differentiation on the Different Cell Types

The above experiments were repeated with different concentrations of freshly prepared DA or OTA, added separately to each differentiation media. Differentiation media for each cell type containing DA or OTA were replaced every two days. The concentrations of DA used were 0.05, 0.1, 0.2, 0.5, 1, 5, and 10 μM, while OTA effects were examined at 0.05, 0.2, 1, and 5 μM. Controls were treated only with the vehicle used to prepare the relevant concentration of chemicals. After 7 days of differentiation process in the presence and absence of DA or OTA, the medium was aspirated, cells were washed, fixed, and immuno-stained for enumeration. The total number of cells (% control) and percentage differentiation [64] of rNSC into astrocytes, oligodendrocytes, and neurons were measured. The RCC was expressed as “total number of cells % control” and has been used as an index of cytotoxicity previously [62,63]. 

### 4.5. Immunochemistry

Fixed cells were incubated for 1 h in blocking buffer (5% goat serum, 1% BSA, 0.1% Triton-X in D-PBS with Ca^2+^ and Mg^2+^). For oligodendrocyte staining, Triton-X was omitted in the blocking buffer, since the A2B5 and Galc markers are a surface antigen. The blocking buffer was removed and cells incubated 18 h at 4 °C with relevant primary antibody diluted in 5% goat serum. The following dilutions were used: For neurons, MAP2 clone M13 monoclonal antibody (4 µg/mL, 1:500), MAP2 clone AP18 monoclonal antibody (1 µg/mL), and Doublecortin monoclonal antibody (10 µL/mL, 1:200); for astrocytes, Anti-GFAP antibody (1:500); for oligodendrocytes, Anti-A2B5 antibody (2 µL/mL) and anti-Galactocerebroside clone mGalc antibody (7 µg/mL); and for rNSC, Anti-Nestin antibody (10 µL/mL). After washing 3× for 5 min with D-PBS containing Ca^2+^ and Mg^2+^, cells were incubated with the relevant fluorescence-labeled secondary antibody (5% serum in D-PBS with Ca^2+^ and Mg^2+^) in the dark at 37 °C for 30–45 min. The cells were washed 3× with D-PBS containing Ca^2+^ and Mg^2+^. Glass slides were removed from chamber compartments, mounted with ProLong diamond antifade reagent with DAPI, and sealed with the coverslip.

### 4.6. Image Analysis

All images were captured using a fluorescence and transmitted light microscope, EVOS FL Color Imaging System equipped with a Sony ICX285AQ color CCD camera. Image analysis was done in 10× and 20× 24-bit color TIFF (1360 × 1024 pixels) images using EVOS FL Color Imaging software with manual assisted cell counting and FIJI ImageJ software. Cells with uniformly stained DAPI nucleus with proper morphology were counted for the quantification of a total number of cells and were compared with the relevant control. Cells stained with specific markers, complete DAPI stained nucleus, and morphology with neurites were considered in quantification. Fluorescent, DAPI, and phase contrast overlapping images were used for percentage cell differentiation quantification. More than 10 randomly selected fields of 200 × 400 μm area were selected from each slide chamber for the percentage cell differentiation quantification, whereas 400 × 800 μm area were selected for total cell quantification. 

### 4.7. Tracing and Measurement of the Axonal Length of the Mature Neurons

Tracing and measurement of the axonal length of the mature neurons were done on rNSC directed into neuron differentiation process in the absence and presence of DA and OTA. After 7 days of treatment, the cells were fixed and stained. Subsequently, axons of treated and control neurons were traced and length measured using Neurite Tracer in the ImageJ software (National Institute of Health, Bethesda, MD, USA). The axons were traced in magenta color in luminous images of controls, in 0.05, 1, and 10 μM of DA- and OTA-treated differentiating neurons.

### 4.8. Statistical Analysis

All the statistical analyses were performed by one-way ANOVA using the IBM SPSS 19 program (IBM, New York, NY, USA). Both the Shapiro–Wilk test and the Kolmogorov–Smirnov test were used to check normal distribution. For concentration response experiments, total cell count, cell differentiation, and axonal length data were normalized within an experiment to corresponding control prior to statistical analysis. Tukey’s HSD post-hoc test was used for a situation with equal sample sizes, and the Scheffe post-hoc test was used for unequal sample size per group. These pairwise comparison tests compare the difference between various parameters of control and the DA- and OTA-treated cells. Non-parametric statistical data were analyzed by one-way ANOVA Kruskal–Wallis H test. All data shown are means ± standard error (SE) in graphs. Statistical significance is given as follows: * *p* < 0.05, ** *p* < 0.01, *** *p* < 0.001 in each experiment legend. 

## 5. Conclusion and Future Perspectives

The present data show that the rNSC monolayer-based system is a reliable assay system for monitoring the neurotoxic effects of DA and OTA. In addition, data show that non-cytotoxic concentrations led to a reduction in the degree of differentiation of rNSC into astrocytes, neurons, and oligodendrocytes. These changes in the cell types and numbers could lead to altered cytoarchitecture and affect processes including cell division, migration, differentiation and cell death, all of which regulate neural development. 

This model system can be used to test the effects of unknown chemicals in conjunction with molecular tools such as transcriptomics, proteomics, and metabolomics to dissect pathways related to the developmental processes.

## Abbreviations

BBB	Blood Brain Barrier
CNS	Central Nervous System
DA	Domoic Acid
DNT	Developmental Neurotoxicology
EGF	Epidermal Growth Factor
FGFb	Fibroblast Growth Factor basic
GFAP	Glial Fibrillary Acidic Protein
MAP2	Microtubule-Associated Protein 2
OTA	Ochratoxin A
PSC	Pluripotent Stem Cells
rNSC	rat fetal Neural Stem Cells
RCC	Relative Cell Count
RT	Room Temperature
T3	Thyroxin hormone
